# Description of lesions in lambs intoxicated with sodium selenite included in intraruminal boluses: Lambs intoxicated with selenium

**DOI:** 10.1002/vms3.1584

**Published:** 2024-08-27

**Authors:** Midori Jocelyn Hernández Serratos, Mireya Juárez Ramírez, Isauro Garrido Fariña, Jorge Luis Tórtora Pérez, Elein Hernández Trujillo, Víctor Manuel Díaz Sánchez

**Affiliations:** ^1^ Faculty of Higher Studies Cuautitlan UNAM Mexico Mexico; ^2^ Faculty of Veterinary Medicine and Zootechnics UNAM Mexico Mexico

**Keywords:** intraruminal boluses, poisoning, selenium

## Abstract

Selenium is an essential micronutrient for ruminants, which participates in the optimal functioning of proteins and enzymes that can combat oxidative stress in the body; however, its toxicity is documented in different species. The objective of this work was to describe macroscopic and microscopic lesions in lambs intoxicated with selenium administered through intraruminal boluses. The main lesions at necropsy were pulmonary oedema; the myocardial surface presented multifocal pale areas; the thyroid and thymus glands were decreased in size, and areas of necrosis, haemorrhage and hyperkeratosis were observed in the reticulum and rumen. At the microscopic level, congestion, haemorrhage, oedema and hyaline membranes were observed in the lung; hepatic congestion, haemorrhage, degeneration and necrosis; degeneration and necrosis of the reticulum mucosa, as well as areas of hyperplasia and hyperkeratosis; myocardial degeneration, necrosis and fibrosis; congestion, haemorrhage, degeneration and renal tubular necrosis; thyroid follicular atrophy and thymic cortical atrophy. This study evidenced the main lesions related to selenium poisoning in lambs supplemented with the mineral through intraruminal boluses.

## Graphical abstract

This study evidenced the main lesions related to selenium poisoning in lambs supplemented with selenium through intraruminal boluses. The main organs affected were the lung, heart, liver, kidney, thymus, thyroid gland and reticulo‐rumen.

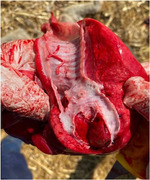

## 1 INTRODUCTION

Poisoning causes illness and death in ruminants, in addition to other clinical problems, such as infectious diseases, trauma or neoplasia. The lack of information on the most common causes of poisoning prevents an effective diagnosis in animals. It has been seen that in sheep and goats, the most common causes of poisoning depend on their geographical location; for example, in Spain, the most common toxicoses are those produced by plants, fungi and metals; in Greece, poisoning by pesticides is the most common, and in Belgium, toxic plants are the most important (Guitart et al., 2010). In Brazil, it is estimated that plant poisoning is responsible for 7.4%–15.8% of cattle deaths; given the predominance of extensive grazing systems (Molossi et al., 2021).

Sheep tend to be more exposed to toxic substances (32.7%) compared to goats (23.0%), due to differences in their eating habits. As sheep graze, they are more likely to consume forage and soil contaminated with toxic agents, unlike goats that can browse and consume leaves taken from trees or bushes (Guitart et al., 2010). However, toxic compounds can also be found in the feed consumed by animals in pens, such as packaged forage, associated with the way it is stored and seasonal changes. Finally, a direct correlation has been observed between production systems and the possibility of poisoning in ruminants. In Spanish, from 1996 to 2001, the total number of deaths due to poisoning was 418, with 275 cases related to extensive systems and 143 to intensive systems in sheep (Guitart et al., 2010; Loh et al., 2020).

Although selenium (Se) is important for ruminants, there is a narrow range between essential levels and its toxicity. This mineral is essential for the functioning of selenoproteins (e.g. glutathione peroxidases and thioredoxin reductases), which have antioxidant activity, detoxification and Se transport and play a key role in thyroid hormone metabolism (Haskins et al., 2017). Currently, selenium requirements in lambs have been set at 0.3 mg/kg DM according to the FDA, in both organic and inorganic form (Novoselec et al., 2022; Pecoraro et al., 2022). However, some previous studies have reported that oversupplementation (>1 mg/kg DM) could have positive effects on production and antioxidant capacity in sheep (Khalil et al., 2023; Mousaie, 2021).

In contrast, its toxicity has also been documented. The first conclusive association of selenium ingestion with poisoning was in horses, due to the observation made by Madison, a US Army physician, who observed that cavalry horses taken to seleniferous areas began to lose vitality, became emaciated and lost weight, and began to lose hair from their manes and tails. Intoxication appears in cattle after ingestion of plants containing approximately 25 ppm Se for several days or weeks, which was called ‘alkaline disease’ (Nogueira & Rocha, 2011).

Acute selenosis in farm animals is the result of oversupplementation in premixes or overdose with oral or parenteral preparations. Acute poisoning may present with sudden death with few clinical signs. However, in most cases, signs of poisoning begin within a few hours to a few days after a toxic dose with the mineral. Clinical signs generally occur in gastrointestinal, cardiovascular and respiratory systems. Motor disorders, ataxia, dark diarrhoea, abdominal pain, bloating, depression, polyuria, pale mucous membranes, hyperthermia, weak and rapid pulse, dyspnea and sometimes cyanosis may be observed. Death is due to circulatory and/or respiratory failure, which usually occurs within 1 or 2 days of exposure. Acute poisoning causes haemorrhagic areas and necrosis in the myocardium, as well as oedema in the lungs (Amini et al., 2011; Raisbeck, 2020). The objective of this work was to describe the histopathological findings in lambs chronically intoxicated with selenium administered through intraruminal boluses.

## 2 MATERIALS AND METHODS

The study was carried out at the Faculty of Higher Studies Cuautitlan of the National Autonomous University of Mexico, 42 Columbia breed lambs were used with approximately 3 months of age and 15 kg of BW, and they were divided into three groups: bolus selenium to which an intraruminal bolus was administered, parenteral selenium supplemented with 0.25 mg/kg of sodium selenite and control (without selenium). The intraruminal selenium boluses had a weight of 8 g and 1.26% sodium selenite (0.1 g) and an approximate size of 24.9 × 14.9 mm^2^.

The lambs remained under the treatments for 3 months, receiving free access food and water, considering that forage in Mexico has selenium levels below 5 ppm (Ramírez‐Bribiesca et al., 2001). To evaluate blood selenium levels, EDTA samples were taken every week, which were evaluated using the atomic absorption spectrophotometry and hydride generator technique (Steen et al., 2008).

**FIGURE 1:**
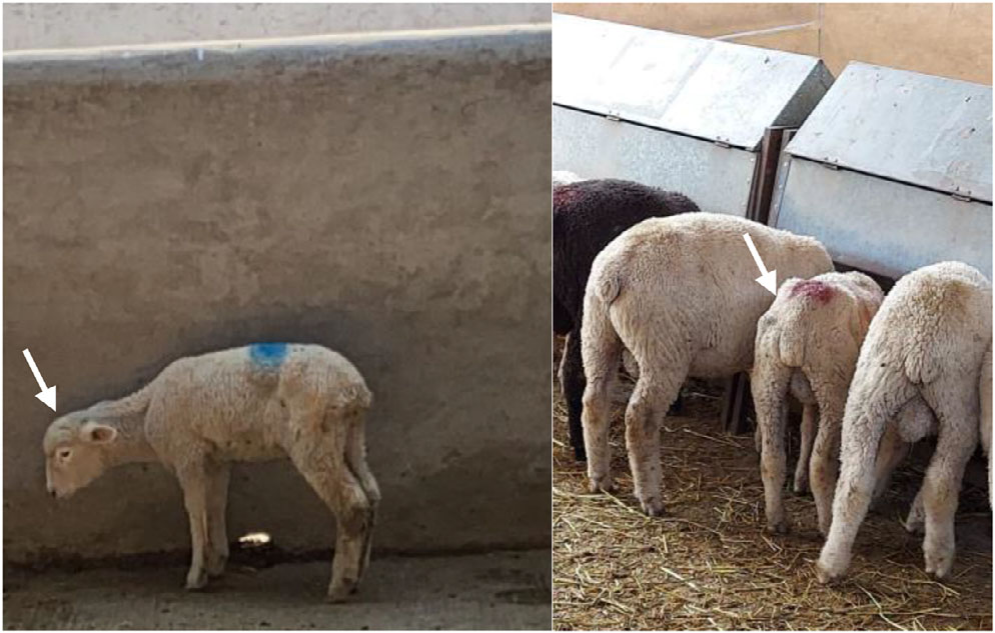
Animals treated with intraruminal boluses with sodium selenite showed weight loss and altered state of consciousness (indicated with arrows).

Approximately 7 days after administration of intraruminal boluses with sodium selenite, four lambs died. The affected animals presented decreased food consumption, decreased body weight, emaciation, apathy and anorexia, alterations in the state of consciousness, and before dying, the sheep showed respiratory difficulty (Figure 1).

The necropsy was performed following the technique described by Cantón and Odriozola (2019). Samples of liver, kidney, lung, rumen, reticulum, heart, thymus and thyroid were obtained, which were fixed in 10% formalin and subsequently embedded in paraffin for processing using the hematoxylin‐eosin technique (Cardiff et al., 2014).

## 3 RESULTS

**FIGURE 2:**
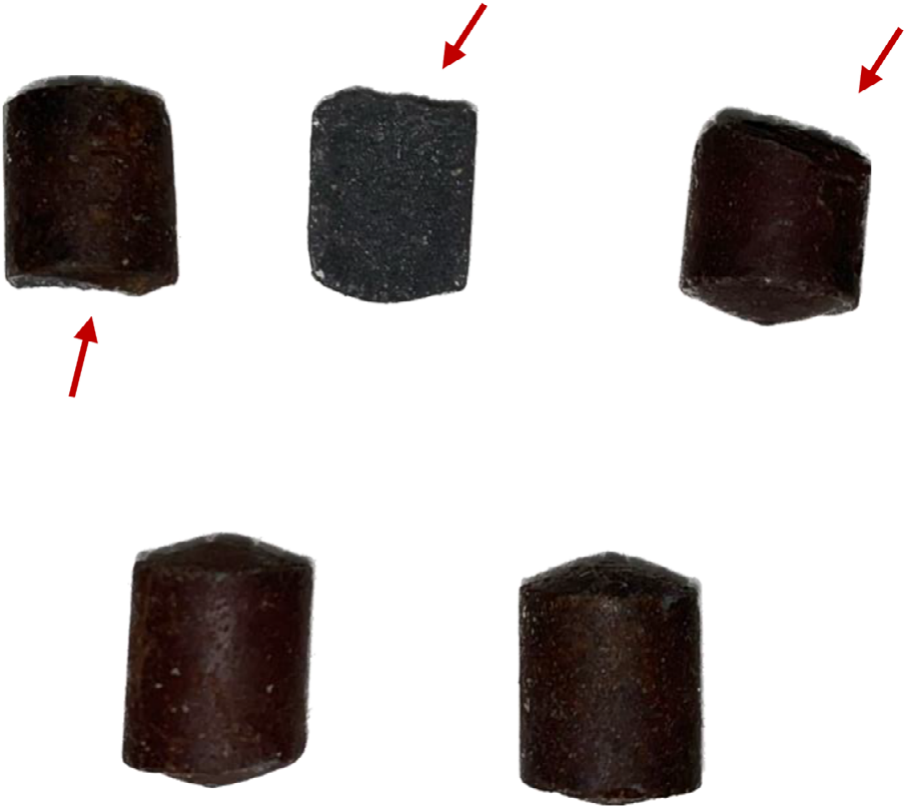
Recovered intraruminal selenium boluses. Fractures on the boluses are indicated by arrows.

During the necropsy, when checking the rumen and reticulum, it was found that the boluses had fractures (Figure 2), altering their integrity, and could have facilitated the abrupt release of the mineral.

**FIGURE 3:**
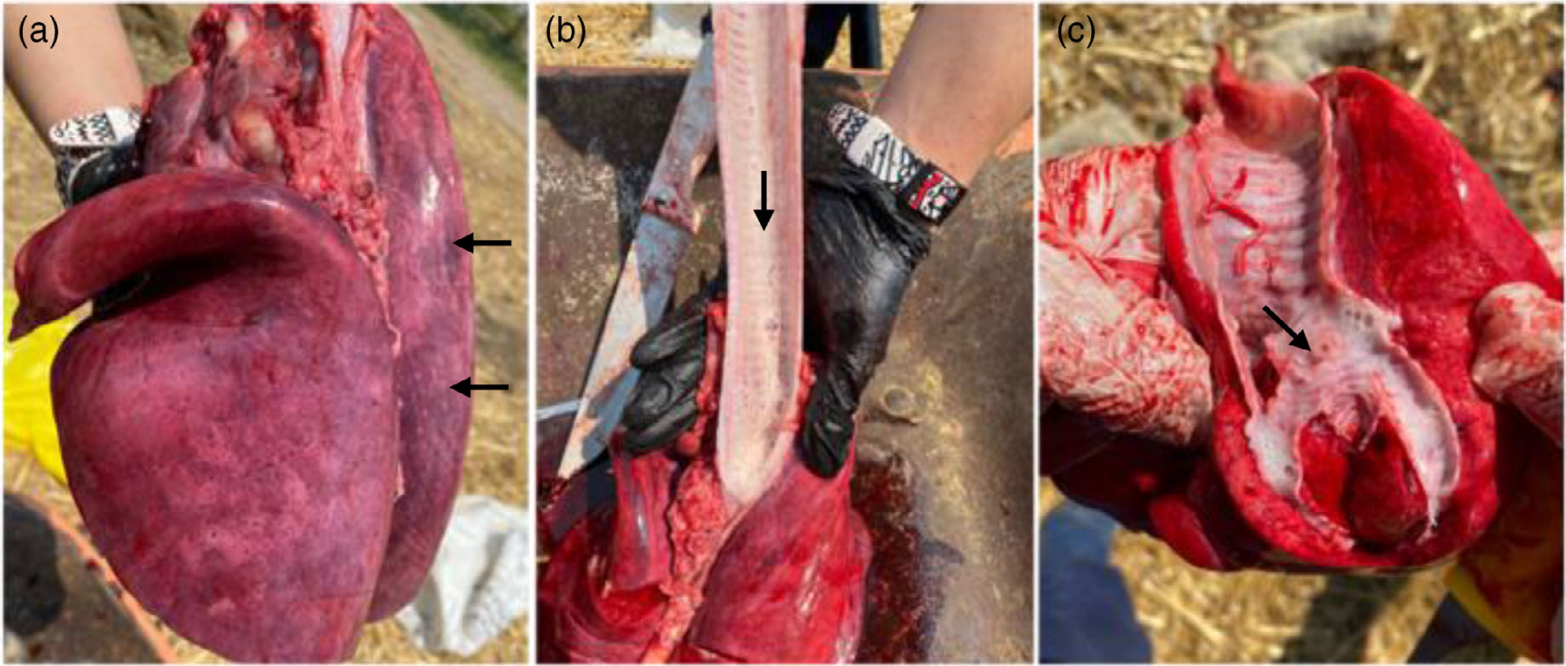
(a) Congestion and pulmonary oedema (indicated in the arrows). (b) Foam on the tracheal surface (indicated by arrows). (c) Foam in tracheal bifurcation (indicated by arrows).

**FIGURE 4:**
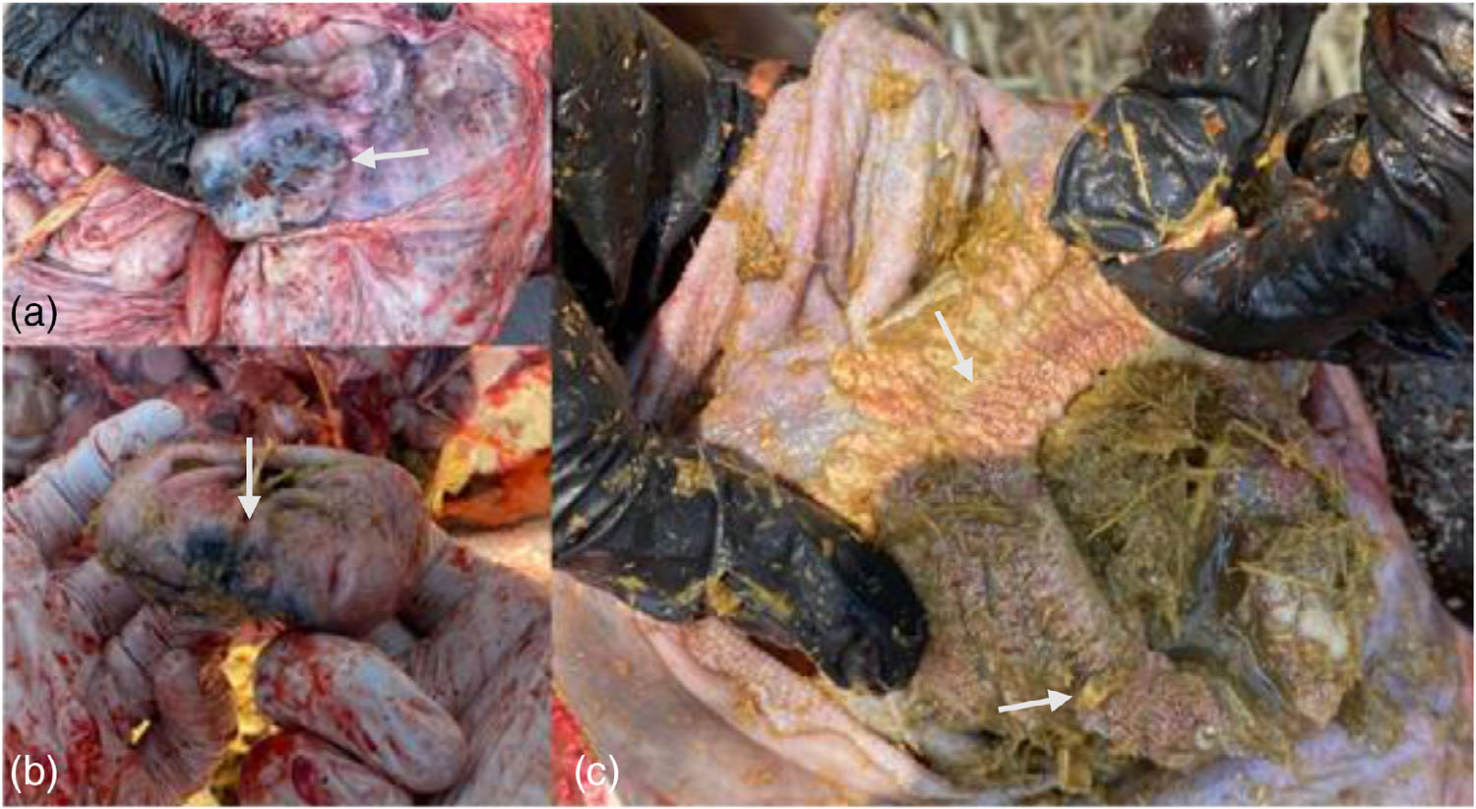
(a and b) Areas of necrosis and haemorrhage in the reticulum. (c) Reticulo‐rumen hyperparakeratosis.

The main findings at necropsy were congestion and pulmonary oedema (Figure 3). The tracheal and bronchial surfaces contained a moderate amount of white foam. Areas of necrosis were observed in the reticulo‐ruminal mucosa, which presented a red‐black colour with loss of papillae (Figure 4).

**FIGURE 5:**
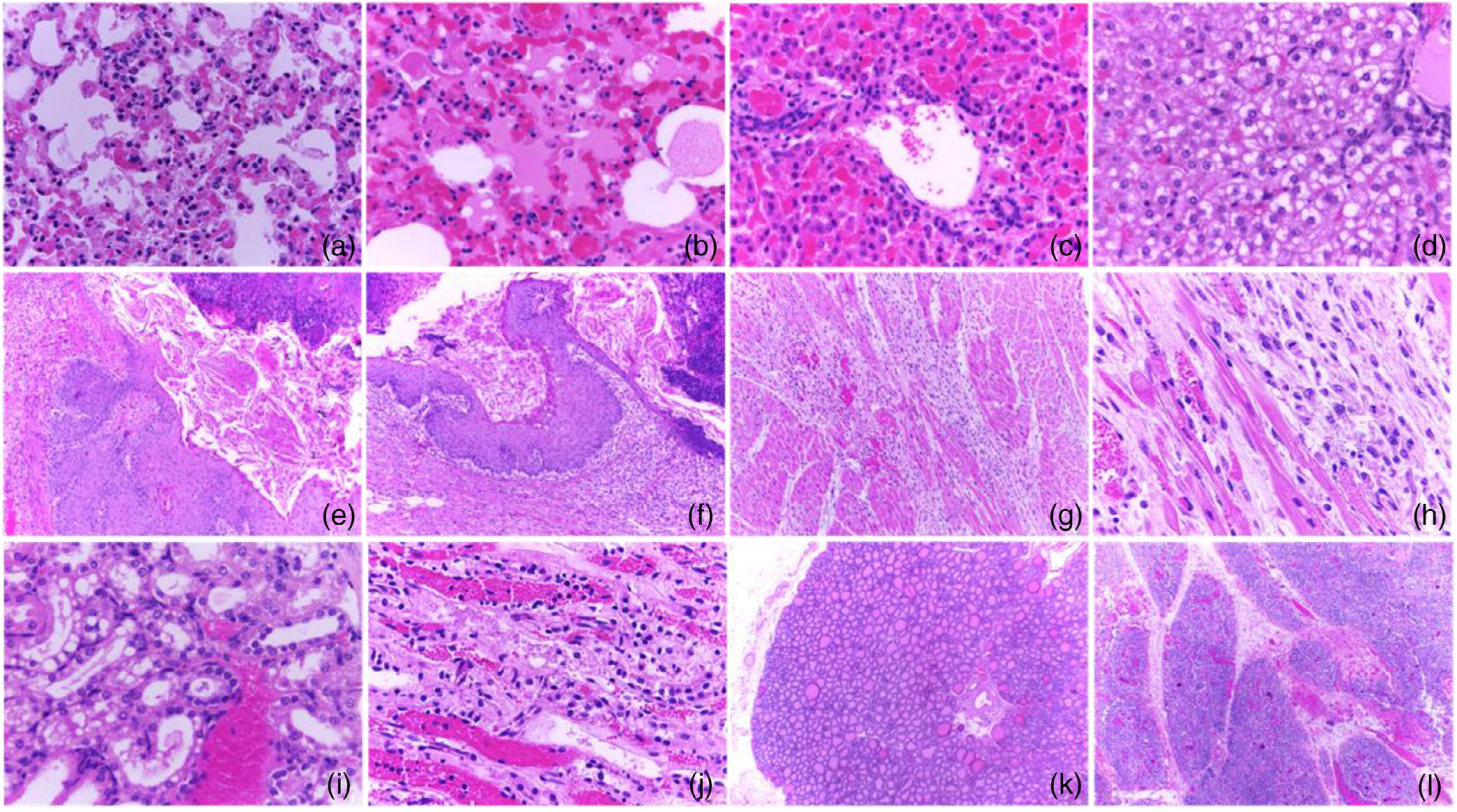
(a and b) Congestion of alveolar capillaries, haemorrhage, oedema and hyaline membranes in the lung. (c and d) Areas of haemorrhage in the liver parenchyma, congested liver sinusoids, degeneration and necrosis of hepatocytes, hepatocellular lipid degeneration. (e and f) Reticulum with areas of necrosis, mucosal degeneration and cellular debris in the lumen, areas of hyperplasia, as well as areas of parakeratotic hyperkeratosis. (g and h) Multiple areas of degeneration and necrosis of cardiac muscle fibres that are replaced and dissected by connective tissue. (i and j) Areas of haemorrhage and congestion in the renal parenchyma, tubular degeneration and necrosis. (k) Atrophy of thyroid follicles. (l) Cortex atrophy in the thymic corpuscles.

Histopathology showed neutrophilic interstitial pneumonia with eosinophilic protein in the alveolar lumen compatible with oedema, as well as congestion of the alveolar capillaries. Severe diffuse pulmonary oedema was observed, as was the presence of hyaline membranes in multifocal alveolar spaces. The alveolar capillaries were markedly congested. In all cases, necrosis and vacuolar degeneration were found in the hepatocytes; areas of haemorrhage and congestion of hepatic sinusoids were also observed. Multiple areas of congestion and haemorrhage were observed in the renal parenchyma, areas of tubular degeneration and necrosis, as well as vacuolar degeneration of the tubular epithelium. In the reticulum, multiple areas were found where the mucosa was observed to be necrotic, accompanied by cellular debris in the lumen. In other areas, the mucosa showed different degrees of hyperplasia, as well as areas of parakeratotic hyperkeratosis. The submucosa was found infiltrated by inflammatory cells, mainly neutrophils. The blood vessels in these areas were congested. Two animals presented severe multifocal cardiomyocyte degeneration and necrosis, where the muscle fibres were observed to be replaced by connective tissue with an abundant number of fibroblasts (Figure 5).

## 4 DISCUSSION

Selenium supplementation in small ruminants has multiple benefits in animal production. This can be done through organic or inorganic mineral salts, which can be incorporated into the diet, water, mineral mixing blocks, injectable solutions or prolonged release forms as intraruminal boluses (Hall et al., 2009; López‐Arellano et al., 2015).

The use of intraruminal boluses reduces animal handling, stress and economic costs. It has been seen that the pharmaceutical form can maintain adequate blood Se values for 3–4 years in animals that consume grasses deficient in this element. However, in Latin America, it is not a common method to supply trace minerals (Gutierrez et al., 2005; León‐Cruz et al., 2020). On the other hand, there are few studies that demonstrate possible selenium poisoning due to the use of intraruminal boluses. However, in a study carried out with lambs where the toxicity of intraruminal boluses with selenium was evaluated, the animals presented tachypnea, a metallic odour in the oral cavity, as well as laminitis (Lopez‐Arellano et al., 2015).

The most important lesions found in the animals were degeneration, necrosis and fibrosis in cardiac muscle fibres, as well as pulmonary oedema, pneumonia and formation of hyaline membranes. This is similar to what was reported by Amini et al. (2011), where in a group of goats that died from selenium poisoning, the most affected organs were the heart and lung, where necrosis of cardiomyocytes was found, as well as foci of haemorrhage. In the lung, congestion and severe oedema were observed, as well as multifocal areas of interalveolar haemorrhage.

Doses of selenium higher than recommended damage antioxidant defences through the depletion of thiols and the generation of reactive oxygen species (ROS). ROS are initiators of uncontrolled oxidation of lipids, proteins and nucleic acids, leading to cellular dysfunction. Moreover, the reduction of glutathione (GSH) acts as a prooxidant in response to oxidative stress induced by selenium (Harisa, 2013), and due to this, the organs that report the most histological lesions are the liver and kidney, as observed in this study.

Different degrees of hepatocellular degeneration, areas of haemorrhage and congestion were observed in the liver, findings consistent with those of Amini et al. (2011), where dead goats presented congestion in hepatic sinusoids; in addition, Johnson et al. (2008) documented hepatocellular degeneration and necrosis in rats following the administration of methyl selenocysteine at doses of 0.5, 1.0 and 2.0 mg/kg/day for 28 days.

Degeneration and necrosis were observed in the kidney, consistent with those described by Smyth et al. (1990) where an experiment was carried out with 12‐week‐old lambs, to which a dose of 5 mg/kg of sodium selenite was administered orally and parenterally to demonstrate the lesions caused by toxicity; these animals showed degeneration in the proximal tubules. Furthermore, He et al. (2014) showed that rats with 8 mg/kg of SeNPs presented necrosis in tubular cells and glomerulitis.

The most important lesions in the thymus were a decrease in the cellularity of the thymic cortex, which agrees with what was reported by Peng et al. (2011), where they showed that the intake of more than 5 mg/kg could cause lesions in the thymus and a decrease in T‐cell subsets. He et al. (2014) also reported a decrease in the thymic cortex in rats supplemented with 8 mg/kg of SeNPs.

It is important to emphasize that in the biodistribution of selenium, the animal organism prioritizes organs, where the liver and kidney are some of the ones that contain the highest levels of the mineral (Zheng et al., 2022), which is probably why the findings in these organs are degeneration and necrosis for both, in addition to several areas of congestion and haemorrhage, unlike the degree of injury found in other organs.

Finally, in the reticulo‐rumen, areas of necrosis, hyperplasia and degeneration of the mucosa were observed, as well as inflammation in the submucosa. These damages could be related to the bolus as a foreign object within the organ. This agrees with the histological damages described by Martín Martel et al. (2021), where they refer to epithelial hyperplasia as the most frequent finding in ruminants, in addition to oedema of the lamina propria. In this work, the authors conclude that these changes could be attributed to chronic friction microtrauma that causes dystrophic epithelial changes, resulting in cellular degeneration and leukocyte infiltration. In addition, lesions can modify the absorption capacity and stimulate inflammation and secondary rumen infection by the organ's microflora. However, in the same way, we could consider the abrupt release of Se, due to the fragmentation of the bolus, as the origin of the lesions in the rumen and reticulum, this agrees with what was observed in the work of Lopez‐Arellano et al. (2015), where the rupture and adhesion of the bolus to the walls of the reticulo‐ruminal mucosa caused coagulative necrosis that extended from the epithelial surface to the submucosa and part of the muscle.

## 5 CONCLUSIONS

In the present work, the main lesions caused by poisoning with selenium supplemented through intraruminal boluses were evident, where the main organs affected were the lung, heart, liver and kidney.

It is of clinical importance to emphasize the toxic potential that even parenteral supplementation of the mineral can have in small ruminants, if the doses are not taken into account.

## AUTHOR CONTRIBUTIONS

**Midori Jocelyn Hernández Serratos**: Investigation; writing – original draft preparation; formal analysis; visualization. **Isauro Garrido Fariña**: Resources; supervision. **Mireya Juárez Ramírez and Jorge Jorge Luis Tórtora Pérez**: Conceptualization; writing – review and editing. **Elein Hernández Trujillo**: Writing – review and editing. **Víctor Manuel Díaz Sánchez**: Methodology; conceptualization; writing – review and editing.

## CONFLICT OF INTEREST STATEMENT

The authors do not report conflicts of interest.

### ETHICS STATEMENT

The authors confirm that the ethical policies of the journal, as noted on the journal's author guidelines page, have been adhered to and the appropriate ethical review committee approval has been received. In accordance with the regulations of the Internal Committee for the Care and Use of Experimental Animals (CICUAE‐FESC C 23_13) of the Faculty of Higher Studies Cuautitlan of the National Autonomous University of Mexico.

### PEER REVIEW

The peer review history for this article is available at https://publons.com/publon/10.1002/vms3.1584.

## Data Availability

Data openly available in a public repository that issues datasets with DOIs.
